# Supplementary material for: Identification of photoperiod-induced specific miRNAs in the adrenal glands of Sunite sheep (Ovis aries)
Source: Front Vet Sci. 2022 Jul 22;9:888207. doi: 10.3389/fvets.2022.888207 (PMC9354845; doi:10.3389/fvets.2022.888207)
Supplement: Supplementary file 1 [file Data_Sheet_1.ZIP › Supplementary file/miRDeep2 file.zip!/miRDeep2 file/Novel_Structure/SPLP42A2/Novel_90.pdf]

Provisional ID	:	NC_019471.2_28143
Score total	:	6.4
Score for star read(s)	:	3.9
Score for read counts	:	-0.8
Score for mfe	:	1.8
Score for randfold	:	1.6
Score for cons. seed	:	
Total read count	:	10
Mature read count	:	7
Loop read count	:	0
Star read count	:	3

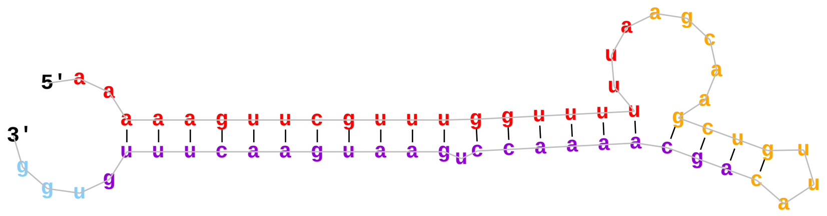

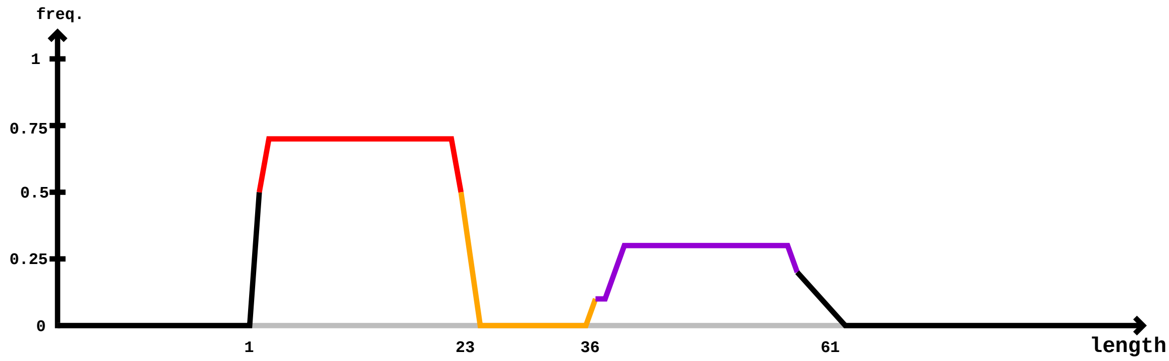

Mature Star

```
5'- auacacuacugggcuggccaaaaaguucguuugguuuuuuaagcaagcuguuacagcaaaaccugaaugaacuuuguggccaacccaagacucuguucccacaggcagaca   -3'    obs
    auacacuacugggcuggccaaaaaguucguuugguuuuuuaagcaagcuguuacagcaaaaccugaaugaacuuuguggccaacccaagacucuguucccacaggcagaca          exp
    .........((((.((((((.(((((((((((((((((........((((...)))))))))).))))))))))).)))))).))))....((((((......))))))..    reads   mm   sample
    ...................aaaaaguucguuugguuuuuu.......................................................................    2       0    seq
    ...................aaaaaguucguuuggGuuuuua......................................................................    3       1    seq
    ....................aaaaguucguuuggGuuuuua......................................................................    2       1    seq
    ......................................................agAaaaaccugaaugaacuuug...................................    1       1    seq
    ........................................................caaaaccugaaugaacuuuUu..................................    1       1    seq
    .........................................................aaaaccAgaaugaacuuu....................................    1       1    seq
```
